# Incidence of groin hernia repairs in women and parity: a population-based cohort study among women born in Sweden between 1956 and 1983

**DOI:** 10.1007/s10029-024-03011-1

**Published:** 2024-03-23

**Authors:** A. Matovu, J. Löfgren, A. Wladis, P. Nordin, G. Sandblom, H. J. Pettersson

**Affiliations:** 1 Mubende Regional Referral Hospital, Plot 6, Kakumiro Road, P.O. Box 4, Mubende, Uganda; 2 Department of Molecular Medicine and Surgery, Karolinska Institutet, Stockholm, Sweden; 3 Linköping University, Linköping, Sweden; 4 Department of Surgical and Perioperative Sciences/Surgery, Umeå University, Umeå, Sweden; 5 Department of Surgery, Södersjukhuset, Stockholm, Sweden; 6 Department of Clinical Science and Education Södersjukhuset, Karolinska Institutet, Stockholm, Sweden

**Keywords:** Groin hernia, Hernia repair, Parity

## Abstract

**Introduction:**

The aim of this study was to evaluate the association between parity and the incidence rate of groin hernia repair in women.

**Method:**

This study was based on two Swedish national registers, the Medical Birth Register (MBR), and the Swedish Hernia Register (SHR). The cohort constituted of women born between 1956 and 1983. Data on vaginal and cesarean deliveries were retrieved from the MBR. The birth and hernia registers were cross matched to identify hernia repairs carried out after deliveries.

**Results:**

A total of 1,535,379 women were born between 1956 and 1983. Among these, 1,417,237 (92.3%) were registered for at least one birth. The incidence rate for Inguinal Hernia Repair (IHR) and Femoral Hernia Repair (FHR) was 10.7 per 100,000 person-year and 2.6 per 100,000 person-year, respectively. Compared with women registered for one delivery, the incidence rate ratio for IHR was 1.31 (95% Confidence Interval: 1.23–1.40) among women registered for two deliveries, 1.70 (1.58–1.82) among women registered for ≥ 3 deliveries. Additionally, the incidence rate ratios were higher 1.30 (1.14–1.49) and 1.70 (1.49–1.95) for FHR among women with two and  ≥ 3 registered deliveries, respectively.

**Conclusion:**

In the present cohort, higher parity was associated with a higher incidence of inguinal as well as FHRs.

## Introduction

### Background

The etiology of groin hernias is multifactorial affecting both men and women [1, 2]. Groin hernias are treated surgically and approximately 10% of the repairs occur in women [2, 3]. This relates to the life-time risk of developing a groin hernia in women estimated to be 3–6% as compared to 27% in men [2, 4]. Some of the factors associated with developing groin hernias in women have been described, for example, increase in age, a low Body Mass Index (BMI), and a positive family history [5–7]. At presentation, groin hernias in women may appear as a lump in the lower parts of the abdomen and the upper thigh [8]. In some cases, they may be occult and present as pelvic or groin pain [9–11].

Pregnancy may be a unique factor in the development of groin hernias in women; however, not fully explored. Tissue reorganization related to hormonal changes and mechanical forces on the anterior abdominal wall during pregnancy may affect the integrity of the supporting muscles and connective tissues around the groin [12, 13]. The resulting tissue weakness poses a risk for groin hernia formation. Femoral hernias are more common in women than men but generally considered to be rare in nulliparous women; however, there are no epidemiological studies confirming this. Incidence rates for inguinal hernia repair (IHR) have been observed to dwindle in some male populations (474–372 between 1989 and 2008) and 168.21–92.10 per 100,000 person-year between 2000 and 2010 probably due to increasing BMI and reduction in smoking but remaining constant for a longer duration in women for unknown reasons [14, 15]. There could be other factors beyond what has been observed that maintain the status of groin hernia repair trends in women. The aim of this study was to evaluate the association between parity and the incidence of groin hernia repair in women. This may support clinical decision-making as regards diagnosis and surgical approaches in relation to parity.

## Methods

**Fig. 1:**
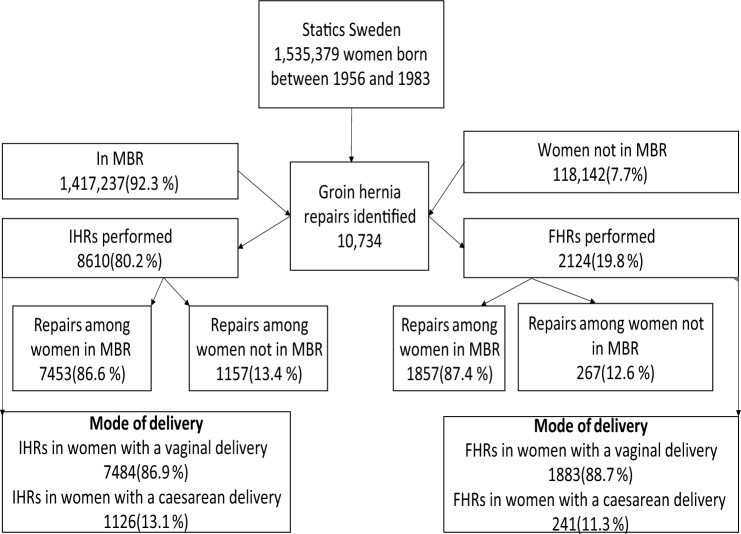
Flow chart for the study incidence. A flow Chart for the study on the incidence of groin hernia repair in women and parity in Sweden. *IHR* inguinal hernia repair, *FHR* femoral hernia repair, *MBR* Medical Birth Register

This study was conducted as a cohort study in Sweden. It was based on two registers: The National Swedish Medical Birth Register (MBR) and the Swedish Hernia Register (SHR). The study cohort included women born between January 1, 1956 and December 31, 1983 (Fig. 1). Data on the total population were retrieved from Statistics Sweden [16]. Statistics Sweden is a government agency that assembles official population statistics showing population size, population changes such as the number of births, deaths, and migration. The MBR was founded in 1973 and includes data on all deliveries in Sweden. It is compulsory for each health care provider to report to the register and the information available is collected from medical records for the prenatal, mode of delivery, and neonatal care [17]. The SHR was established in 1992. It contains data on groin hernia repairs performed in patients aged 15 years and older. The purpose of the register is to survey the development of hernia surgery in Sweden in terms of methods of repair, waiting time, care policies, and results. It also provides a base for local evaluation of treatment outcomes, enabling epidemiological studies and supporting randomized controlled trials. The register reports on the methods of repair, anesthesia, anatomical circumstances, and the general information about the surgeon, patient, and the surgical procedure. Nearly all hernia repairs performed in Sweden are registered in the SHR [18, 19]. Data on deliveries distinguishing between vaginal and cesarean deliveries were retrieved from the MBR. A cesarean delivery was defined as a history of at least one cesarean section regardless of when and how many. The MBR was cross matched with the SHR to identify hernia repairs carried out after childbirth.

Considering previous estimates, in relation to the study cohort we presumed that the majority of women born in Sweden between 1956 and 1983 to have had a first delivery relatively from 19 years of age [20]. According to a previous Swedish study, the likelihood of developing a groin hernia requiring surgery is greatest in women who are 40 years and older [21]. Using these two approximations of age at first childbirth and age to develop a groin hernia, we analyzed data for women born between January 1, 1956 and December 31, 1983. We used this study cohort since the likelihood of having given birth as well as reaching the threshold for developing a groin hernia and the possibility of having undergone groin hernia repair as determined from the MBR and SHR should be greatest within this age interval. Ethical approval was obtained from the Ethical review authority, Sweden (2021-03090).

### Inclusion criteria

The inclusion criteria were women 19 years old and above, born between January 1, 1956 and December 31, 1983, having undergone a groin hernia repair, and registered in the SHR. There were no exclusion criteria for this study.

### Primary endpoints

The primary endpoint was to determine the volume of inguinal and femoral hernia repair (FHR) among women born between 1956 and 1983.

### Statistical methods

To estimate the incidence rates for IHR, we calculated the numerator in the incidence rates as the sum of the number of women in the MBR and SHR with an IHR for each year. The denominator, i.e., the person-year, was calculated as the sum of person-year for women in MBR with hernia repair according to the SHR, person-year for women not in MBR but with a hernia repair according to the SHR, and person-year for women not in MBR and not in the SHR and without hernia repair. Person-years indicate the number of persons in this study and the time frame each individual remains in this study. It is a measure in cohort studies for exposure to the risk of developing a disease.

The person-year was calculated as the time from the year of birth to the year when the hernia was operated or the end of follow-up period in 2022, whichever occurred first. The calculations for FHR incidence rates were done in the same way as for the IHRs.

Analysis was done in SPSS version 28 and we used open Epi Version 3.01 to calculate confidence intervals for the incidence rate ratio.

### Ethical approval

Ethical approval was obtained from the Ethical review authority, Sweden (2021-03090).

## Results

In this study, 1,535,379 women born between 1956 and 1983 and residing in Sweden were included. Of these, 1,417,237 (92.3%) gave birth in reference to the MBR. A total of 10,734 groin hernia repairs were identified from the SHR. Of these 8610 (80.2%) were IHRs and 2124 (19.8%) were FHRs. The mean incidence rate for IHR was 10.7 per 100,000 person-year (Table 1) and 2.6 per 100,000 person-year for FHR (Table 2), respectively. Increased IHR and FHR incidence rate ratios were observed in respect to parity.

**Table 1:** Women born in Sweden between 1956 and 1983, those in the MBR and incidence rates of IHR

Total number of women born between 1956 and 1983	Total number of women in the MBR	Total number of women not in the MBR^a^	Total number of women without an IHR and not in the MBR	Total number of IHRs among women in the MBR	Total number of hernia repairs among women not in the MBR	Mean incidence rate for IHR per 100,000 person-year
1,535,379	1,417,237	118,142	116,985	7453	1157	10.7

*MBR* Medical Birth Register

^a^ Refers to the women who had a groin hernia repair but could not be found in the Medical Birth Register. It is possible that these women migrated to Sweden after giving birth or emigrated after hernia repair and had childbirth out of Sweden

**Table 2:** Women born in Sweden between 1956 and 1983, those in the MBR and incidence rates for FHR

Total number of women born between 1956 and 1983	Total number of women in the MBR	Total number of women not in the MBR^a^	Total number of women without a FHR and not in the MBR	Total number of FHRs among women in the MBR	Total number of FHRs among women not in the MBR	Mean incidence rate for FHRs per 100,000 person-year
1,535,379	1,417,237	118,142	117,875	1857	267	2.6

^a^ Refers to the women who had a groin hernia repair but could not be found in the Medical Birth Register. It is possible that these women migrated to Sweden after giving birth or emigrated after hernia repair and had childbirth out of Sweden

### Inguinal hernia

**Fig. 2:**
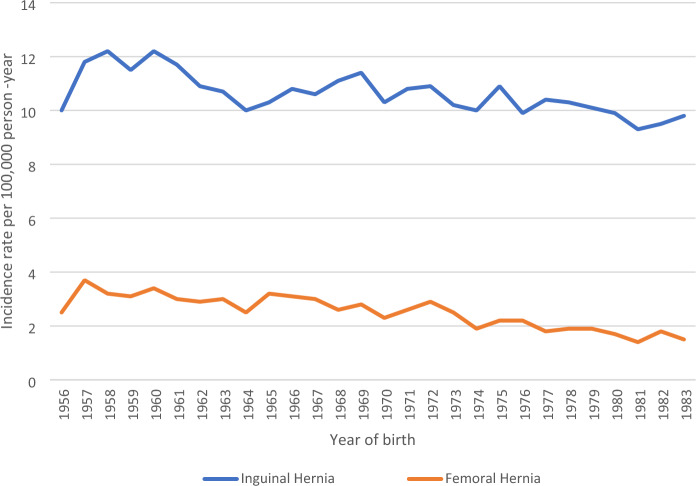
Incidence rate for inguinal and FHRs in women followed from year of birth until 2022. *Women born in 1956 had longer follow-up compared to those born in 1983. The incident rate trends are persistent with slight decreases between 1956 and 1983

Of the 8610 IHRs, 7453 (86.6%) repairs were encountered among women in the MBR. A total of 1157 (13.4%) IHRs were identified among women who were not in the MBR. The incidence rate for IHRs among women in the study population was 10.7 per 100,000 person-year and it was distributed in a similar way for women born between 1956 and 1983 (Fig. 2) although with a decreasing trend over the study period.

A total of 1126 (13.1%) IHRs occurred among women who had a cesarean delivery. The incidence rate of IHR among women with cesarean delivery was 8.7 per 100,000 person-year. On the other hand, 7484 (86.9%) IHRs were among women who had a vaginal delivery and the incidence rate of IHR in this group was 11.1 per 100,000 person-year. Compared to vaginal delivery, the incidence rate for cesarean delivery was lower, incidence rate ratio 0.78 (95% Confidence Interval, 0.74–0.83) (Table 3).

**Table 3:** Incidence rate ratios of IHR in association with mode of delivery and parity levels

Variable	Number of IHRs	Person-year	Incidence rate per 100,000 person-year	Incident rate ratio (95% CI)
Mode of delivery				
Vaginal delivery	7484	67,354,049	11.1	*Reference*
Cesarean delivery	1126	12,921,207	8.7	0.78(0.74–0.83)
Parity				
Parity 1	1204	16,168,457	7.45	*Reference*
Parity 2	3546	12,921,207	9.45	1.31(1.23–1.40)
Parity ≥ 3	2703	21,393,088	12.63	1.70(1.58–1.82)
Not in MBR^a^	1157	6,329,555	18.28	2.45(2.26–2.66)

*MBR* Medical Birth Register

^a^ Refers to the women who had a groin hernia repair but could not be found in the Medical Birth Register. It is possible that these women migrated to Sweden after giving birth or emigrated after hernia repair and had childbirth out of Sweden

There were varied parity levels among women with IHR. The lowest incidence rate for IHR was found among women with one delivery (7.4 per 100,000 person-year). Comparing to women with one delivery, the incidence rate ratios were higher for women with two deliveries 1.31 (2.26–2.66) and among those with three or more deliveries 1.70 (1.58–1.82). Women not registered in the MBR had an incidence rate ratio of 2.45 (2.26–2.66) (Table 3).

### Femoral hernia

A total of 2124 (19.8%) FHRs were encountered among women in the study population. Of these, 1857 (87.4%) repairs occurred among women in the MBR and 267 (12.6%) repairs were among women who were not in the MBR. The incidence rate for FHR in the study population was 2.6 per 100,000 person-year and was evenly distributed for women born between 1956 and 1983 (Fig. 2). A decreasing trend was observed from 1956 to 1983.

A total of 241 (11.3%) FHRs occurred among women who had a cesarean delivery. The incidence rate for FHR among women with a cesarean delivery was 1.87 per 100,000 person-year. On the other hand, 1883 (88.7%) FHRs occurred among women who had vaginal delivery and the incidence rate was 2.79 per 100,000 person-year. Comparatively, cesarean deliveries had a higher incidence rate for FHRs as compared to vaginal deliveries with an incidence rate ratio of 1.50 (1.31–1.71) (Table 4).

**Table 4:** Incident rate ratio of FHRs in association with mode of delivery and parity

Variable	Number of FHRs	Person-year	Incidence rate per 100,000 person-year	Incidence rate ratio (95% CI)
Mode of delivery				
Vaginal delivery	1883	67,400,731	2.79	*Reference*
Cesarean delivery	241	12,921,207	1.87	1.50(1.31–1.71)
Parity				
Parity 1	300	16,168,457	1.86	*Reference*
Parity 2	800	36,384,156	2.42	1.30(1.14–1.49)
Parity > = 3	676	21,393,088	3.16	1.70(1.49–1.95)
Not in the MBR^a^	267	6,376,237	4.19	2.26(1.91–2.66)

*MBR* Medical Birth Register

^a^ Refers to the women who had a groin hernia repair but could not be found in the Medical Birth Register. It is possible that these women migrated to Sweden after giving birth or emigrated after hernia repair and had childbirth out of Sweden

In this evaluation, FHRs were distributed differently according to the different parity levels. The lowest incidence rate for FHR was found among women with one delivery (1.86 per 100,000 person-years). Compared with women with one registered delivery, the incidence rate ratios were higher for women with two deliveries 1.3 (1.14–1.49) and for three or more deliveries 1.70 (1.49–1.95). Women not registered in the MBR had an incidence rate ratio of 2.26(1.91–2.66) (Table 4).

## Discussion

### Key results

In this cohort study, the incidence rate for IHR was 10.7 per 100,000 person-year among women born in Sweden between 1956 and 1983. Higher parity and vaginal deliveries were associated with a higher incidence rate for IHRs. The incidence rate of FHRs was 2.6 per 100,000 person-year. A higher parity and cesarean delivery were associated with a higher incidence rate for FHR.

### Interpretation

The results reflect outcomes on the follow-up of women born between 1956 and 1983. It was more likely that women born between 1956 and 1983 were registered in the MBR if they delivered the first child in Sweden and entered in the SHR if they had had a groin hernia repair during the study period. This enabled us to determine the incidence rates and incidence rate ratios reported in this study.

The 10.7 incidence rate of IHRs per 100,000 person-year and the 2.6 incidence rate for FHRs per 100,000 person-year found in this study were evenly distributed over the study period. Our study had a longer follow-up duration than previous studies. A 20-year follow-up (1989–2008) found a higher incidence rate for IHR among adult women, 44 per 100,000 person-year that remained constant for over 15 years duration before declining [14]. Among women, parity may have contributed to the even distribution of the IHR and FHR observed in our study.

The increased incidence rate ratios of IHR and FHR in relation to parity may point to history of deliveries as a risk factor for hernia development. We observed that there is an increase in the incidence rate ratios of IHR as parity increases, 1.31 (95% Confidence Interval, 1.23–1.40) for parity two and 1.70 (1.50–1.82) for parity ≥ 3 and the increasing incidence rate ratios for FHR, 1.30 (1.14–1.49) for parity two and 1.70 (1.49–1.95) for parity ≥ 3. These findings show that parity one is associated with a 30% increase in groin hernia repair rates and parity three is associated with a 70% increase in the groin hernia repair rates. The non-overlapping confidence intervals in IHR, as well as the FHR incidence rate ratios, may mean an association between the levels of parity, IHR, and FHR. Pregnancy has hormonal as well as mechanical effects on the inguinal and femoral canal. There may be related derangement and weakening of the supporting connective tissues and the extracellular matrix around the inguinal and femoral canals resulting into hernia formation [22–25]. This is coupled with increased intra-abdominal pressure and connective tissue exertion which are major factors in the etiology of hernias [26]. In addition, abdominal muscle distension and decreased contractility effect in the postpartum period have also been demonstrated [13]. It has been observed that there are more inguinal hernias in men than women and vice versa for femoral hernias which may be due to anatomical differences [2, 27–29]_._ However, it could also be due to parity.

Cesarean deliveries demonstrated a lower incidence rate ratio for IHR, incidence rate ratio 0.78(0.74–0.83), and vice versa for FHR, incident rate ratio 1.50(1.31–1.71). The reasons behind this finding in our study is not clear. There was a 28% reduction in IHR incidence among women who had a cesarean section unlike in women who had a FHR, where we see a 50% increase in the incidence of repair. It is possible that surgeons may have been reluctant to offer hernia repair to women with previous lower abdominal surgery. It is also possible that women who have undergone a cesarean operation are more reluctant to undergo groin hernia repair or that a lump in the groin is more difficult to detect. However, a previous report suggested that abdominal operations are not associated with inguinal hernias in females [30]. On the other hand, in comparison to men, lower midline abdominal surgery has been associated with inguinal hernias more so after radical retropubic prostatectomy [31–33]. This finding needs more investigation.

### Limitations

The registry data did not take into consideration immigration and deaths. Women who immigrated from Sweden and those who died were not censored. Between 1956 and 1983, Sweden experienced more immigration than emigration. This implies that the person-year in the denominator is higher which in turn leads to an underestimation of the absolute incidence rates for inguinal and FHRs that we present. On the other hand, lack of data on deliveries for some of the women before immigration to Sweden affects the incidence rate ratios in relation to parity and mode of delivery. Some women may have undergone a hernia repair in Sweden but we cannot create an association with parity and mode of delivery for lack of those specific data in the MBR. Hernia repair without added person-years means a higher incidence rate but when you compare this with immigration and deaths; then most likely the reported incidence rates are underestimated.

Additionally, of the total population of women in Sweden aged 65 years or more in 2022 (*n* = 1,142,384), 167,702 (14.7%) were born abroad [16]. Hence, we do not have data on deliveries for these women unless they immigrated before childbearing age. Reliable data on nulliparity would have given a better understanding of the relationship between parity and risk of groin hernia repair. However, we do not have these data. We have used parity one as our reference point instead. This study did not adjust for other factors like BMI, smoking, positive family history, socioeconomic status, and education levels which could impact on the occurrence of groin hernias and the incidence of groin hernia repair. Some of these have been dealt with in previous studies.

### Generalizability of the results

There seems to be a greater risk for groin hernias in women after two or three deliveries than after one delivery. There is high prospect that parity increases the incidence of groin hernias in women also in other populations. The distribution of other risk factors for developing groin hernia in women are also different between different populations. To what extent this impacts the association between hernia development and childbirth is not known.

## Data Availability

Data are available on request from the corresponding author in anonymous form.
